# Workplace friendship, employee well-being and knowledge hiding: The moderating role of the perception of Chaxu climate

**DOI:** 10.3389/fpsyg.2022.1036579

**Published:** 2022-11-29

**Authors:** Peixu He, Jun Wang, Hanhui Zhou, Chi Zhang, Qiyuan Liu, Xin Xie

**Affiliations:** ^1^Business School, Huaqiao University, Quanzhou, China; ^2^Kedge Business School, Domaine de Luminy, Marseille, France

**Keywords:** workplace friendship, employee well-being, knowledge hiding, perception of Chaxu climate, social exchange theory

## Abstract

In recent years, knowledge hiding has become a hot topic in the field of organizational behavior because of its great harm. However, relevant studies have focused only on the negative interpersonal antecedents of knowledge hiding but neglected the inhibition effect of positive informal relationships on the behavior. To fill this gap, the current study develops a moderated mediation model to investigate how and when workplace friendship has a negative impact on knowledge hiding. Drawing on social exchange theory (SET), we propose that workplace friendship inhibits knowledge hiding through the mediating role of employee well-being, with the Perception of Chaxu climate acting as a boundary condition. Using data from a two-wave time-lagged survey of 279 employees in China, the results show that workplace friendship has a negative impact on knowledge hiding behavior. Specifically, workplace friendship inhibits knowledge hiding by satisfied employee well-being, i.e., workplace friendship has a positive impact on employee well-being, while employee well-being has a negative impact on knowledge hiding. Perception of Chaxu Climate moderates the indirect effect, as the level of employee’s Perception of Chaxu climate rises, the direct effect of workplace friendship on employee well-being is stronger, so as the indirect effect of workplace friendship on knowledge hiding. This article explores the mechanisms affecting employee knowledge hiding from a new interpersonal perspective of workplace friendship. It is enlightened that firms should pay attention to the management of workplace friendship, provide employee with opportunities to establish workplace friendship while providing proper guidance on the direction of workplace friendship and improving the quality of it, in order to promote employees’ happiness perception and organizational knowledge management ability.

## Introduction

The knowledge-based view emphasizes that the enterprise is a knowledge-processing system that takes employees as the carrier to realize knowledge sharing in various ways to gain competitive advantages (Lakshman et al., 2021). Therefore, knowledge sharing has become a crucial way to promote employees’ knowledge creation and sustainable development of enterprises (Batistič and Poell, 2022). However, most employees are unaware of the importance of knowledge sharing and may even consider knowledge as a limited resource that needs to be hidden (Qin et al., 2021). For example, a survey by Peng (2013) showed that 46 percent of Chinese employees admitted that they had knowledge hiding behavior to their colleagues when their colleagues asked them for “knowledge help,” suggesting that knowledge hiding is widespread in companies (Jiang et al., 2019; He et al., 2021). Knowledge hiding refers to the behavior of employees who deliberately choose to conceal and retain knowledge for certain purposes when their colleagues ask them for help (Connelly et al., 2012). This behavior is prone to serious consequences for the organization and can hinder the effective sharing and utilization of knowledge in the workplace (Garg and Anand, 2020; Butt, 2021). And previous studies have also shown that it can damage interpersonal relationships, reduce creativity, and increase mistrust among employees, leading to a vicious circle (Černe et al., 2014). Therefore, understanding more about the causes of knowledge hiding behavior and inhibiting it has great significance for the common development of employees and organizations.

As a typical interactive behavior among organizational members, the influence of interpersonal factors on knowledge hiding has attracted the interest of many scholars (Zhao and Xia, 2019). Relevant studies have particularly focused on the negative interpersonal antecedents of the behavior, such as workplace ostracism, workplace negative gossip, and employee mistrust (Connelly et al., 2012; Zhao et al., 2016), expecting to understand the mechanism of the occurrence of knowledge hiding for better interventions (He et al., 2020), but neglecting the important role of positive coworker relationships in inhibiting this behavior. Among interpersonal relationships, workplace friendship, which has a double goal of being instrumental and valued, plays an extremely critical role in employees’ behavioral attitudes and intentions (Enwereuzor et al., 2022). As a non-exclusive interpersonal relationship between organizational members involving trust, commitment, mutual affection, and common interests (Berman et al., 2002), workplace friendship not only triggers positive emotion, cognition, and organizational citizenship behavior among employees but will also inhibit the occurrence of negative individual behavior (Scott et al., 2018; Guohao et al., 2021; Enwereuzor et al., 2022) For example, Zhuang et al. (2020) found that creating a friendly working environment for employees in international tourist hotels can reduce their production deviance, political deviance, property deviance, and personal aggression. However, although scholars have argued that workplace friendship among colleagues can inhibit the occurrence of negative behavior in organizations, the existing research does not provide a clear answer as to whether knowledge hiding, a type of interpersonal deviant behavior of employees, is negatively influenced by workplace friendship and what specific mechanism exists between them. Thus, it is especially valuable to explore the mechanism of workplace friendship’s influence on employees’ knowledge hiding behavior.

Furthermore, social exchange theory states that the similarities in individual characteristics drive both members to engage in a variety of high-quality exchange activities (Cropanzano et al., 2017). Since workplace friendship is a spontaneous and informal interpersonal relationship established by employees (Pillemer and Rothbard, 2018), knowing each other’s role expectations, individuals on the team will help each other’s role expectations by providing relevant resources based on friendship relations (Hsu et al., 2019). Therefore, we consider that reciprocal behaviors triggered by workplace friendship may satisfy organizational members’ needs for job fulfillment and self-actualization and, in turn, positively affect employee well-being. Employee well-being refers to the overall quality of employees’ experience and efficacy at work (Grant et al., 2007), which usually includes three dimensions: work, psychology, and life. It can reflect the individual’s well-being in various ways and is closely related to issues such as work engagement, life satisfaction, and occupational health (Zheng et al., 2015). Previous research has shown that employee well-being as a healthy psychological mechanism has become an essential antecedent factor in the field of organizational behavior to explain employees’ behavior and attitudes (Ahmed et al., 2020). Employees with high levels of well-being usually show a strong tendency to exhibit positive behaviors, they are willing to share their knowledge resources and expect to achieve a win-win situation between themselves and the organization through efficient knowledge management (Ali et al., 2021). Indeed, while the management literature emphasizes the importance of employee well-being in prompting better individual and organizational outcomes, few studies have investigated the well-being of Chinese employees and the behavioral tendencies of these employees to hide their knowledge in the context of workplace friendship. Therefore, to fill the above-mentioned gaps, this study incorporates employee well-being into the exploration of mediating mechanisms based on moral cleansing theory, in order to further clarify the intrinsic mechanisms of workplace friendship and employees’ knowledge hiding behavior.

**Figure 1:**
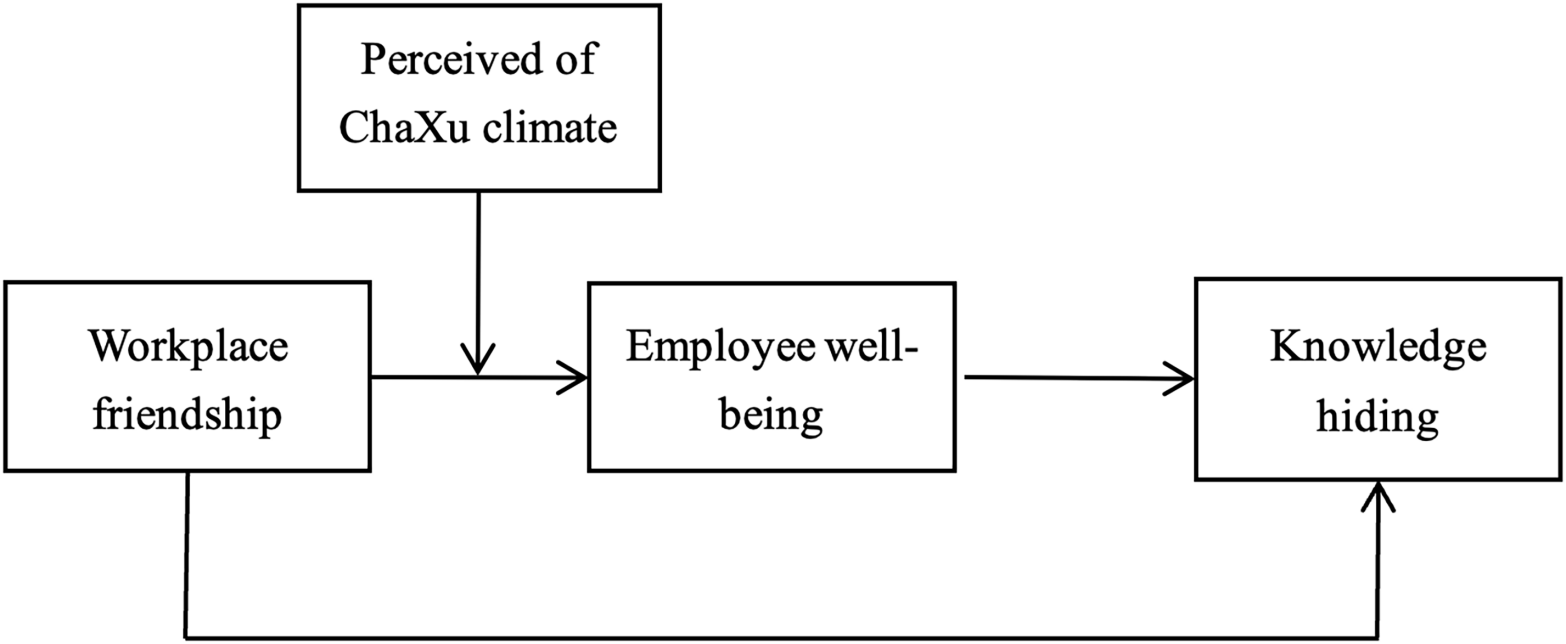
Theoretical model.

In addition, according to social exchange theory, the quality of interpersonal exchange is often influenced by many factors such as individual characteristics, values, and cultural backgrounds of employees (Cropanzano et al., 2017), Especially under the influence of Confucianism’s “guanxi” and “circle” culture, the cognition and behavior of Chinese employees will have obvious characteristics of the Chaxu climate (He et al., 2017). This means that in workplace friendships, individuals also follow the social norms of proximity and inferiority, and habitually categorize themselves as “insiders” or “outsiders” on the basis of the difference in their relationship with the leader, which has a destructive effect on the formation of reciprocal relationships among the organization/group (Song and Guo, 2022). As described by Shen et al. (2019), Among the traditional cultures, the Chaxu social structure with the value of “kinship and respect” is prevalent in Chinese companies, not only forming a Chaxu climate mainly characterized by the “circle culture” within the organization (Shen et al., 2019), but also plays an important role in shaping the mindset and behavior of Chinese employees (Chen et al., 2014). When employees perceive more pronounced differential treatment from their leaders, namely the more intense perception of a Chaxu climate, they may experience a stronger sense of “marginalization” (Chen et al., 2014) and even lose trust in other members (Wang et al., 2017), which in turn hinders knowledge exchange and sharing within the team (Peng and Zhao, 2011). However, few studies have explored the impact of employees’ perception of Chaxu climate on their own behavior (Shen et al., 2019). Therefore, we intend to introduce social exchange theory to explore the negative effect of workplace friendship *via* employee well-being on their knowledge hiding, and to examine the moderating effect of employees’ perception of Chaxu climate in this process. The theoretical model is shown in Figure 1.

This study contributes to the existing literature in the following ways. Firstly, this study extends the research related to knowledge hiding by identifying new antecedent factors. In past studies on the interpersonal antecedents of knowledge hiding, most tend to focus on negative factors or formal organizational relationships, while ignoring the influence of informal and positive interpersonal relationships. Therefore, this study contributes to the research concerning reducing and inhibiting knowledge hiding by exploring the negative influence of workplace friendship on employees’ knowledge hiding behavior based on the perspective of social exchange, shifting the focus of interpersonal antecedents of knowledge hiding from formal to informal relationships and from driving factors to hindering factors. Secondly, by identifying employee well-being as a mediating mechanism between workplace friendship and knowledge hiding behavior, this study not only enriches the literature on the positive organizational utility of workplace friendship, but also expands the research relevant to employee well-being. In fact, although scholars believe that workplace friendship can inhibit the occurrence of negative behaviors in organizations, existing studies do not provide a clear answer to the question of whether knowledge hiding, as a type of interpersonal deviant behavior of employees, is inhibited by workplace friendship and whether employee well-being plays a mediating role in it. Therefore, our study goes beyond previous literature and empirically examines the role of workplace friendship in promoting employee well-being and how employee well-being is associated with knowledge hiding. Finally, this study identifies that employees’ perceptions of Chaxu climate negatively moderate workplace friendship through the effect of employee well-being on knowledge hiding, expanding the research perspective on the Chaxu climate. The current study mainly investigates the Chaxu climate as an antecedent or outcome variable, but few scholars have analyzed the moderating effect from the perspective of boundary conditions. Our study complements the research on the Chaxu climate by illustrating the moderating role in “workplace friendship-employee well-being-knowledge hiding.”

## Literature review and hypotheses

### Workplace friendship

Interpersonal relationships in organizations include work relationships (e.g., leader/subordinate) and workplace friendship (Berman et al., 2002; Mao and Hsieh, 2017). According to Berman et al. (2002), workplace friendship is defined as a non-exclusive interpersonal relationship in an organization that involves trust, commitment, mutual affection, and shared interests or values, which is often divided into two dimensions: friendship opportunity (i.e., allowing employees to establish informal relationships with each other) and friendship quality (i.e., the extent to which the relationship is maintained between the two members to the friendship and the psychological gains it brings; Nielsen et al., 2000). Pillemer and Rothbard (2018) state that, unlike other positive work relationships, workplace friendship has the following four core characteristics: first, workplace friendship is voluntary relationship and employees establish it through autonomous rather than imposed choices (Yan et al., 2021). Second, workplace friendship is an informal relationship. Unlike other role relationships determined by formal organizational hierarchies, it relatively lacks “standard rituals or terminology” to limit role expectations (Choi and Ko, 2020). Third, workplace friendship is characterized by communal norms or expectations that one will provide support based on his/her needs rather than just reciprocity (Pillemer and Rothbard, 2018). Fourth, workplace friendship is driven by social affective goals (e.g., intimacy, trust) and was designed to meet the affective needs of employees (Hood et al., 2017).

These show that workplace friendship has stronger affective features than other work relationships and has significant impacts on employees’ attitudes, behaviors, and performance. Specially, in the aspect of attitudes, high workplace friendship usually leads to stronger interpersonal networks for individuals. They are more likely to receive supports from friends when completing tasks, and the sense of belonging can be substantially enhanced. As a result, they typically show high psychological security (Cao and Zhang, 2020), high job satisfaction (Fasbender and Drury, 2022) and affective commitment (Guohao et al., 2021). Regarding behavior, workplace friendship can effectively promote employees’ organizational citizenship behavior (Scott et al., 2018) and reduce deviance (Zhuang et al., 2020). These are because of two reasons, one is that workplace friendship facilitates employees’ access to more supportive resources which lead to an increased sense of organizational support (Xiao et al., 2020), and the other is that workplace friendship can stimulate positive reciprocity and develop positive affections and cognitions (Enwereuzor et al., 2022). Finally in terms of job performance, some studies have found that workplace friendship positively impacts job performance (Choi and Ko, 2020), while others have argued that individual in a friendship may experience more frequent and intense socio-affective disruptions because of social interactions, which leads to a decreased individual job performance (Pillemer and Rothbard, 2018). Still others have found that multiple workplace friendships may be a mixed blessing. While providing and restoring resources fostered by multiple relationships benefits employees’ job performance, this also weakens as employees suffer from emotional exhaustion and depletion of personal resources (Methot et al., 2016).

Based on social exchange theory, individuals have the need to reward the benefits of the relationship in order to keep a positive relationship they have received in social interactions, i.e., individuals maintain the positive efficacy by conducting behaviors that can benefit each other. It is the “mutual needs” which lead to social exchange behavior (Cropanzano et al., 2017). According to this principle of “positive reciprocity,” when an employee’s personal needs are met, he/she will reciprocate to the friends. In practice, employees’ good friendship means there are strong affective connections between employees and colleagues, and between employees and the firm. With the improvement of the interactions, employees will receive stronger organizational supports and happiness perceptions, which will encourage them to show a “positive reciprocity” attitude to reward the organization. They will not only engage in pro-social behaviors (e.g., knowledge sharing) that directly benefit the firm (Enwereuzor et al., 2022), but also pay more attention to their own deviance and willingly reward the firm by inhibiting the deviance (Zhuang et al., 2020), including knowledge hiding. As suggested by Batistič and Poell (2022), if a colleague’s behavior shows his/her trust and concern, the employee will perceive a positive image of the colleague and feel an overall sense of responsibility or desire to reciprocate the behavior. This can lead to a reciprocal cycle, thus reducing knowledge hiding. Therefore, when faced with knowledge requests from colleagues, employees who have great friendships are more likely to response the requests by knowledge assistance in order to reward the colleagues/firm, i.e., they may show a lower intention of knowledge hiding.

### Workplace friendship and knowledge hiding

According to Connelly et al. (2012), knowledge hiding is defined as “the phenomenon of employees deliberately deceiving, misleading and concealing knowledge when facing colleagues’ requests for knowledge assistance,” which contains three specific forms: evasive hiding, playing dumb and rationalized hiding. As a kind of knowledge withholding behavior, knowledge hiding is not equivalent to the opposite of knowledge sharing (He et al., 2021), but the differences lie in the followings: firstly, knowledge not being shared is not entirely due to the individuals lack of willingness to share knowledge, but may also be a result of the non-deliberate ability factors, such as lack of knowledge or unfamiliarity with relevant knowledge; whereas knowledge hiding is the employees’ deliberate and purposeful hiding of the requested knowledge despite having the knowledge. Second, knowledge sharing is a proactive behavior, but knowledge hiding is a reactive behavior, i.e., knowledge hiding is a response to others’ knowledge requests (Burmeister et al., 2019). As described by Connelly et al. (2012), knowledge hiding is more of a bilateral interpersonal activity. Therefore, interpersonal relationships within the organization/team may play a critical role in the occurrence of it (Butt and Ahmad, 2019). For example (He et al., 2020), found that leader-member exchange relationship (LMX) and supervisor-subordinate guanxi (SSG) inhibit the occurrence of knowledge hiding through the mediating effect of psychological security. Similarly, as a part of the organization’s informal interpersonal relationships, workplace friendship may also have an impact on employees’ knowledge hiding.

As mentioned above, workplace friendship is a voluntary friendship formed by employees, which is not only a communal norm of equal reciprocity, but also creates conditions for them to receive instrumental and affective support (Mao et al., 2012). Except for promoting reciprocal behaviors among employees, we believe that workplace friendship may also inhibit individuals’ knowledge hiding. Specially, we focus on the characteristic of workplace friendship that is “socio-affective goal-driven.” Employees may perceive a stronger organizational culture of trust and reciprocity when the organization has a cordial communication climate, encourages informal communication among members, and provides conditions that enable the development of informal interpersonal relationships (i.e., more friendship opportunities). Along with further enhancement of such relationship (i.e., stronger friendship quality), employees will receive more supportive resources, such as psychological and affective resources (Xiao et al., 2020). These could meet employees’ affective needs. Further, based on social exchange theory, employees are activated with reciprocal psychology as their affective needs are met (Yan et al., 2021), and they expect to reward their firms and colleagues by performing more pro-organizational behaviors (Berman et al., 2002). Because of such sense of reciprocity and belonging (He et al., 2020), the respondent is generous and free to provide his/her knowledge resources when faced with others’ knowledge requests (Xia et al., 2021), rather than providing deceptive information (evasive hiding), making excuses (rationalized hiding) or pretending to be an ignorant (playing dumb).

Furthermore, social exchange theory suggests that individuals will assess the risks in the exchange and decide which attitude or behavior to adopt based on the result of the assessment (Zhang et al., 2022). Just as the core characteristic of workplace friendship-“common norms”-shows close and equal relationships among employees, friends care about each other’s real needs and are willing to provide affective and resource support to the other, even when they receive nothing in return (Lee and Chon, 2020). From this point, the common norms in workplace friendship like an invisible psychological contract, which includes long-term trust, dedication, and affective exchange among friends. And this creates a nearly “zero-risk” social exchange situation for employees, resulting in the reinforcement of their reciprocal beliefs. Therefore, when individuals are confronted with knowledge requests from others, especially from their friends, they are more willing to engage in knowledge assistance in a positive and reciprocal manner rather than knowledge hiding. On the one hand, friends trust each other so that employees expect to be rewarded in the future (Bock et al., 2005). On the other hand, employees can obtain other resources from friends to compensate for the losses of knowledge resources (Pillemer and Rothbard, 2018). In summary, we propose the following hypothesis:

*H1*: Workplace friendship has a significant negative effect on knowledge hiding.

### The mediating role of employee well-being

Employee well-being refers to employees’ evaluation of the overall quality of their positions and work experience. According to Zheng et al. (2015), employee well-being is mainly composed of three dimensions: life well-being (employees’ satisfaction with their own life as a whole), work well-being (individuals’ satisfaction with work-related factors, especially the positive emotions they experienced) and psychological well-being (individuals’ psychological experience and satisfaction representation of their work and personal life). In essence, it is the employee’s overall perception of their satisfaction with work and life, and the subjective experience expressed based on this perception. This comprehensive assessment not only includes whether the employee is competent in the current job, whether the requirements of the job or the environment are compatible with the employee’s values and ambitions, but also reflects the affective experiences and states of satisfaction experienced by the employee. More importantly, it also reflects the quality of employee’s social interactions with other members in the workplace (Zheng et al., 2015). Good social interactions increase communication and cooperation among members and satisfy employees’ needs in terms of job fulfillment and affections, leading to higher well-being. While high-quality well-being has an impact on their attitudes and behaviors (Sharma et al., 2016), making them willing to participate in interpersonal interactions, show more caring and kindness to others (Ryff and Singer, 2008), and even share their own unique resources (Wang et al., 2017). Based on these, we examine the mediating role of employee well-being in the negative effect of workplace friendship on knowledge hiding.

We believe that social interactions in the workplace profoundly affect employee well-being (Agneessens and Wittek, 2008). According to social exchange theory (Cropanzano et al., 2017), those employees who have good friendships with their colleagues usually share similar values and career goals. This similarity leads them to trust each other (Requena, 2003) and is helpful for them to develop a positive exchange relationship. Subsequently, mutually supportive behaviors and exchanges between members will be triggered so that employee well-being can be enhanced. The nature of workplace friendship, such as equal reciprocity and shared exchange, means that good friends usually care about the working conditions and performance of employees (Mao et al., 2012), and they are willing to be sincere and open to mutual resource assistance to meet each other’s role expectations (Hsu et al., 2019). Thus, individuals with high workplace friendships are usually more competent in their positions and achieve their career goals. And when an employee achieves such job satisfaction, the level of work well-being and psychological well-being he feels is enhanced (Guerci et al., 2022). Secondly, keeping a good friendship also represents a stable and special affective tie with others because of its affective exchange function (Zhang et al., 2022). The better the friendship between members, the smoother the affective exchange between them. When employees encounter difficulties or frustrations at work, friends can help them to release stress through appropriate affective care and communication (Pillemer and Rothbard, 2018) so that they will experience positive affections such as a sense of security and comfort (Hsu et al., 2019). As described by Zhang et al. (2018), work and family are interdependent, which means employees’ relevant experiences at work can spill over into the home sphere. From this point of view, workplace friendship can not only lead to positive work experiences for individuals, but can also penetrate into other areas outside of work at the same time, creating a positive life experience that increases their life well-being and psychological well-being (Hsu et al., 2019). In summary, workplace friendship can contribute to the overall improvement of employee well-being by stimulating individuals’ work well-being, life well-being, and psychological well-being.

Furthermore, we believe that better well-being leads to optimistic behavioral intentions (Chiu et al., 2013; Woo et al., 2015) and inhibits employees’ negative behavioral tendencies. Specifically, employees who hold positive psychological traits and enjoy their current jobs usually care about the needs of others and willing to offer advice or help to them, which induce communications and interactions among members (Zheng et al., 2015). In such situations, knowledge is not viewed by them as a private unique competitive element, but as a shared resource that can support friends and enhance their own well-being (He et al., 2020). When such employees are confronted with knowledge requests from their peers, they believe that sharing the needed knowledge is not only effective in helping the peers to achieve their work goals, but also allows themselves to feel the joy of helping others which can satisfy their affective needs. Although knowledge providers may still analyze the implicit costs of implementing knowledge sharing, worry about their status in the organization or fear ridicule for providing ineffective knowledge (Anand et al., 2021), having a high quality of well-being does mean that the employees had gain job achievement or affective satisfaction, so they are able to accept the risks related to knowledge sharing. Therefore, we hypothesize that employee well-being can significantly inhibit the occurrence of knowledge hiding.

Putting all into consideration, we suggest that when there is a good friendship between employees, their well-being about the current jobs can be significantly increased so that they are more willing to provide the needed knowledge, which reduce or inhibit their knowledge hiding. Accordingly, the following hypothesis is proposed in this paper:

*H2*: The relationship between workplace friendship and knowledge hiding is mediated by employee well-being, i.e., workplace friendship positively impacts employee well-being, and employee well-being negatively impacts their own knowledge hiding.

### The moderating role of perception of Chaxu climate

Although employees may be grateful or reciprocate to the organization or colleagues for workplace friendship, it is worth noting that not all employees will react to workplace friendship and employee well-being to the same extent. As discussed above, the interpersonal preferences and processes of employees in Chinese firms are not stable and are highly susceptible to the influence of traditional cultural factors, especially Confucianism. Confucianism emphasizes that when individuals interact with others, they should follow the social norms of Chaxu, “respect who they should respect” and “intimate who they should intimate,” which is in line with the spirit of “Li” and “Yi” in traditional culture. Besides, individuals should conform the spirit of “Li” in Confucianism by treating others differently based on Chaxu. Further, “Ren,” “Yi,” and “Li” are consisting of Chaxu in the Chinese interactions, which has significant impacts on Chinese employees’ thinking and behaviors, even on Chinese firm’s operations and management (Liu et al., 2009). For example, Chinese leaders do not treat their subordinates equally, but habitually divide them into “insiders” or “outsiders” and treat them differently in terms of affective attachment and resource allocation (Peng and Zhao, 2011). Once this differentiated treatment is extended to the team level, the Chaxu climate that mainly characterized by a “circle culture” is gradually formed within the team (Shen et al., 2019).

Chaxu climate refers to the difference in relational distance between members around the resource controller in firms (usually the leader; Peng and Zhao, 2011), which is essentially an experience of differential treatment by the leader. Based on the degree of relational heterogeneity, the leader divides the members into those who are at the core of the relationship and those who are at the margins. This could lead to the employee’s perception of Chaxu climate (Song and Guo, 2022). When there is a strong Chaxu climate in an organization, employees’ perception of differential treatment by leaders are stronger, and the perception of marginalization leads to negative psychological experiences of neglect and indifference, making employees more likely to develop cognitive and behavioral biases (Huang et al., 2018). In addition, as mentioned above, employees depend on “mutual needs” to shape their social exchange relationships and behaviors with friends. Different perception of Chaxu climate obviously affects the rate of social exchange and interfere with the quality of reciprocal relationships between members and the willingness to engage in extra-role behaviors (Beck and Schmidt, 2013). Therefore, we hypothesize that employee perception of Chaxu climate may be an important boundary condition in the negative effect of workplace friendship *via* employee well-being on their knowledge hiding and may play a negative moderating role.

Specifically, if employees perceive a stronger Chaxu climate, they may perceive large differences in the quantity and quality of resources that different members are able to receive from the leader. This can lead to a strong sense of differential treatment by the leader (Dhiman and Maheshwari, 2013). Here, the leader-centered working relationship plays a dominant role in all working relationships within the team, including friends (Song and Guo, 2022). In this situation, due to the different perception of Chaxu climate, the members in a friendship will focus more on the difference between their own identity and that of others (Neubert et al., 2008), and may naturally divide the friends into insiders and outsiders. This inevitably disrupts interpersonal exchange among friends (Beck and Schmidt, 2013), and harm employee well-being. For outsiders who are far from the leader, the higher perception of Chaxu climate leads to stronger perceptions of distrust and unfairness, triggering their stronger jealousy to the insiders (Dhiman and Maheshwari, 2013). This induces negative feelings such as hostility and resentment toward insiders from outsider, while employee well-being may be damaged as a result. Moreover, during processing of Chaxu situational information, the insiders can be hostile and defensive for consolidating their status. They may not only ignore or treat the outsiders with indifference, but also lose reciprocity (Beck and Schmidt, 2013). This may undermine employees’ belonging and affective needs, as well as decrease employee well-being (Gregersen et al., 2016). Thus, for all workplace friends, employee well-being will decrease as the perception of Chaxu climate increases.

Conversely, if employees perceive a weak Chaxu climate, they may think that the quality and quantity of resources received by different members from leaders are same, which forms the perception of fairness climate. The mutual commitment and trust among friends can be further enhanced (Shen et al., 2019), and psychological or affective perceptions can be improved so that employee well-being can be increased (Hsu et al., 2019). Additionally, as described by Chen et al. (2014), employees’ perception of Chaxu climate is often seen as a “barometer” of the relationship between subordinates and supervisors. The lower the perception of Chaxu climate, the closer the relationship between employees and supervisors, and the closer the distance between themselves and resources (Wang et al., 2017). From this perspective, employees with lower perception of Chaxu climate will experience a stronger sense of organizational support, which satisfies employees’ belongingness and affective needs. As a result, employee well-being is enhanced (Craig and Kuykendall, 2019). Therefore, the positive impact of workplace friendship to employee well-being will be facilitated when employees have a lower perception of Chaxu climate. So, we propose that:

*H3*: Employee perception of Chaxu climate negatively moderates the effect of workplace friendship on employee well-being, and vice versa.

By integrating H1, H2, and H3, we expect that employees’ perception of Chaxu climate plays a negative moderating role in the indirect effect of “workplace friendship on knowledge hiding *via* employee well-being.” Specifically, based on social exchange theory, workplace friendship inhibits knowledge hiding, and employee well-being mediates the effect. However, considering the relational distance of individuals to their supervisors, the mechanism may be influenced by employees’ perception of Chaxu climate. The positive effect of workplace friendship on employee well-being may be decreased when employees have a high perception of Chaxu climate. In this situation, the inhibitory effect of workplace friendship on knowledge hiding through employee well-being will be weakened. On the contrary, the positive effect of workplace friendship on employee well-being may be enhanced when employees have low perception of Chaxu climate. Further, the inhibitory effect of workplace friendship on knowledge hiding through employee well-being may also be enhanced as a result. Therefore, we hypothesized that:

*H4*: Perception of Chaxu climate moderates the indirect effect of workplace friendship on knowledge hiding through employee well-being, i.e., the indirect effect is weaker when the perception of Chaxu climate is higher and vice versa.

## Materials and methods

### Participants, procedures, and methods

In this study, questionnaires were used to collect data for testing the proposed research hypothesis. Firms in several provinces of China were selected with front-line employees as the sample. To reduce common method biases, we adopted a two-wave employee self-assessment questionnaire. According to Podsakoff et al. (2012), the time lag in data collection at different time phases should not be too long nor too short. In general, an optimal choice of time lag is 2–4 weeks (Wang et al., 2022). Therefore, we collected data in two different time points separated by 2 weeks. Specifically, We first explained the project to the head of the human resource management department of each enterprise. Once we obtained permission from the HR heads, they in turn helped us distribute the survey links and follow up with the data collection. Before the questionnaire, the respondents were selected randomly, asking whether they agreed to join the survey voluntarily and informing them that they could withdraw from the survey at any time.

At Time 1, we collected demographic information as well as the workplace friendship and asked them to rate their employee well-being, perceived of ChaXu climate levels. At Time 2, 2 weeks later, the employees were asked to evaluate their knowledge hiding behavior. Afterwards, the questionnaires distributed on the two different occasions were matched according to the questionnaire codes. Due to the travel constraints linked to the COVID-19 pandemic, it was inconvenient for us to have face-to-face contact with the survey participants in 2022, so we used Wenjuanxing, a platform^1^ that is widely used in China to carry out online surveys. By asking the employees to use the last four digits of their mobile phone numbers as the questionnaire references, we were able to match the participants at the two different time points. A total of 300 employees completed both online surveys. After excluding invalid samples, such as those that could not be matched between the two time points and those with incomplete information (e.g., involving too many missing items), we obtained 279 valid matching samples of subordinates, which represented a response rate of 93%.

### Measures

The scales used in this study were authoritative with good reliability and validity that are widely used by scholars. In order to ensure the consistency of the translated scales with the original scales, we strictly followed the principle of translation procedure. The original scale was translated (English to Chinese) and back-translated (Chinese to English) to generate the Chinese version of the measurement scale. Specifically, two postgraduates with outstanding academic research achievements in human resource management and high English proficiency were invited to translate the original English scale into Chinese, and then two other postgraduates with similar levels were invited to translate the above Chinese scale back into English, and two rounds of English-Chinese translation were conducted according to the above steps. In addition, a small pre-test was conducted before the survey, more precisely, the initial questionnaire pre-survey of this study was conducted in several industries in Fujian Province involving service, tourism, manufacturing and other industries, and the survey objects were 92 front-line employees of enterprises, which is concentrated in young and middle-aged employees, with medium and low educational backgrounds. After conduct CITC, reliability/validity analysis and CFA on the collected pre-survey data, the analysis results showed that all scales such as Workplace friendship, Perception of Chaxu climate, Employee well-being and Knowledge Hiding had good reliability and validity. A 5-point Likert scale was used for both the pre-test and the two-wave survey.

### Workplace friendship

The scale developed by Nielsen et al. (2000) was used, and nine items were finally retained according to the revision of Chinese scholar Jianmin Sun, such as “I have the opportunity to get to know my colleagues in my company; my company encourages communication among employees; I feel I can trust other employees in my company; I have formed strong friendships at work, etc.” (Cronbach’s *α* = 0.92).

### Perception of Chaxu climate

The scale developed by Luo et al. (2016) was used, containing 11 items in 3 dimensions: mutual dependence, partial treatment, and trusted role. Typical items include “I feel that leaders treat subordinates very differently throughout the organization” and so on (Cronbach’s *α* = 0.94).

### Employee well-being

This variable was measured using a scale developed by Zheng et al. (2015), including 3 dimensions of life, work, and psychological well-being, each with six items, for a total of 18 items, such as “Most aspects of my life are close to my dream” (Cronbach’s *α* = 0.93).

### Knowledge hiding

We used the scale developed by Connelly et al. (2012) which contains 12 items in three dimensions. Typical items include “I agreed to help but did not really intend to do so” and “I would say I did not know, even though I did” (Cronbach’s *α* = 0.91).

### Controls

Previous scholars have pointed out that employees’ age, gender, educational background, and experience affect their attitudes and behaviors (Zhou et al., 2019). Therefore, we treated these factors as control variables. Specifically, we asked participants to report age and years of experience data, while educational background was measured in four categories: “less than college, college, bachelor’s degree, master’s degree and above.”

## Results

### Confirmative factor analysis

In this study, AMOS 24.0 was used to conduct confirmative factor analysis through five research variables of workplace friendship, perception of Chaxu climate, employee well-being, and knowledge hiding. The results are shown in Table 1. Among the models, the fit indices of the 4-factor model met the standards with *χ*^2^/df = 2.522, CFI = 0.880, TLI = 0.868, IFI = 0.880, and RMSEA = 0.074. And the 4-factor model fitted better than the 3-factor model, 2-factor model, and single-factor model, indicating good discrimination among the four variables.

**Table 1:** The results of confirmative factor analysis.

Model	*χ*^2^/df	RMSEA	CFI	TLI	IFI
4-factor model + CMV: WF; CX; EW; KH; CMV	2.417	0.071	0.905	0.900	0.906
4-factor model: WF; CX; EW; KH	2.522	0.074	0.880	0.868	0.880
3-factor model: WF + CX; EW; KH	5.809	0.132	0.618	0.583	0.620
2-factor model: WF + CX + EW; KH	6.508	0.141	0.561	0.523	0.564
Single-factor model: WF + CX + EW + KH	7.624	0.154	0.471	0.426	0.474

WF is workplace friendship, CX is perception of Chaxu climate, EW is employee well-being, KH is knowledge hiding, CMV represents common method variance. “+” represents the combination of factors.

### Common method biases test

Although the two-wave self-assessment questionnaire used in this study can reduce common method biases (Podsakoff et al., 2003), we followed Podsakoff et al. (2003) to assess common method biases by introducing an unmeasured latent factor, i.e., common method variance (CMV) in the confirmative factor analysis to enhance the reproducibility of the findings. The results were shown in Table 1, compared with the fit of the 4-factor model, the fit indicators of the 4-factor model + CMV (*χ*^2^/df = 2.417, RMSEA = 0.071, CFI = 0.895, TLI = 0.877) not vary by more than 0.02, which shows that there is no significant common method biases in this study.

### Descriptive statistics and correlation analysis

The results of descriptive statistics and correlation analysis for each variable are presented in Table 2. The results indicate that workplace friendship has a positive correlation with employee well-being (*r* = 0.629, *p* < 0.01), and in terms of the correlation coefficient between workplace friendship and knowledge hiding (*r* = −0.296, *p* < 0.01), workplace friendship may have a direct negative effect on knowledge hiding. Additionally, it also shows that the coefficient between employee well-being and knowledge hiding is (*r* = −0.487, *p* < 0.01). Therefore, we can preliminarily conclude that there may be some negative relationships between workplace friendship, employee well-being and employee’s knowledge hiding.

**Table 2:** Descriptive statistics and correlation analysis.

Variables	Gender	Age	EB	Experiences	Workplace friendship	Perception of Chaxu climate	Employee well-being	Knowledge hiding
Gender	–							
Age	−0.178**	–						
EB	−0.029	−0.025	–					
Experiences	−0.260**	0.722^**^	−0.074	–				
WF	0.046	0.167**	0.060	0.219**	–			
CX	−0.092	−0.126*	0.079	−0.137**	−0.141^**^	–		
EW	−0.075	0.149*	0.214**	0.224**	0.629^**^	0.114	–	
KH	0.035	−0.017	−0.058**	−0.110	−0.296**	−0.221^**^	−0.487**	–
Means	1.53	2.03	2.98	2.92	4.069	2.826	4.133	2.053
SD	0.500	0.717	0.578	0.880	0.798	1.122	0.644	0.815

The numbers of sample is 279; ** represents *p* < 0.01, * represents *p* < 0.05, two-tails test. EB is educational backgrounds. Age, gender, educational backgrounds, and experiences are control variables.

### Hypothesis test

Main effect test. To test the main effect H1, workplace friendship and knowledge hiding were first set as independent and dependent variables separately. We can see from Table 3 that the workplace friendship positively affects employee knowledge hiding (M6, *β* = 0.293, *p* < 0.001) based on the introduction of control variables (gender, age, education backgrounds, and experiences). Thus, H1 was supported by the data.

**Table 3:** The mediating effect.

Variables	Employee well-being				Knowledge hiding				
	M1	M2	M3	M4	M5	M6	M7	M8	M9
Gender	−0.007	−0.096	0.015	−0.065	0.003	0.056	−0.049	−0.002	−0.034
Age	−0.037	−0.042	−0.030	−0.032	0.154	0.157*	0.138	0.131	0.122
EB	0.259**	0.204***	0.250***	0.188***	−0.099	−0.066	−0.077	0.064	0.069
Experiences	0.197***	0.088	0.210**	0.102*	−0.197**	−0.131	−0.225**	−0.037	−0.098
WF		0.487***		0.505***		−0.293***			−0.038
EW								−0.628***	−0.566***
CX			0.760*	0.114***			−0.172***		−0.132**
R^2^	0.092	0.434	0.105	0.471	0.011	0.086	0.062	0.232	0.257
ΔR^2^	0.105	0.445	0.121	0.482	0.025	0.102	0.079	0.246	0.276

The numbers of sample is 279; ** represents *p* < 0.01, * represents *p* < 0.05, two-tails test. EB is educational backgrounds. Age, gender, educational backgrounds, and experiences are control variables.

Mediating effect test. As shown in Table 3, workplace friendship had a significant positive effect on employee well-being (M2, *β* = 0.487, *p* < 0.001), while employee well-being (M8, *β* = −0.628, *p* < 0.001) has a significant negative effect on knowledge hiding. In addition, using Bootstrapping repeated sampling 5,000 times analysis, workplace friendship was analyzed by putting workplace friendship and employee well-being, perception of Chaxu climate and knowledge hiding into the regression equation at the same time, we found that both the direct effect (*β* = −0.293, *p* < 0.01) and indirect effect (*β* = −0.312, *p* < 0.01) of workplace friendship on knowledge hiding reached the significance level. Meanwhile, the indirect effect of employee well-being in workplace friendship → employee well-being → knowledge hiding was (*β* = −0.312, *p* < 0.001) with 95% CI of [−0.483, −0.085] and 95% confidence interval excluding 0. Therefore, the indirect effect of employee well-being reached the significance level, but its direct effect (*β* = −0.157, *p* > 0.05) with 95% CI was [−0.328, 0.014] with 95% confidence interval including 0, thus its direct effect did not reach the significance level and employee well-being had a significant fully mediated effect between these two variables. Therefore, H2 was supported by the data (Table 4).

**Table 4:** The results of bootstrap.

Type	Paths	Effects	SE	Boot 95%CI		Relative effect
				LL	UL	
	WF → HK	−0.293**	0.061	−0.413	−0.174	–
The independent mediating effect of EW	WF → HK	−0.157	0.087	−0.328	0.014	33.5%
	WF → EW → HK	−0.312***	0.102	−0.483	−0.085	66.5%

The numbers of sample is 279; ** represents *p* < 0.01, * represents *p* < 0.05, two-tails test. EB is educational backgrounds. Age, gender, educational backgrounds, and experiences are control variables.

**Figure 2:**
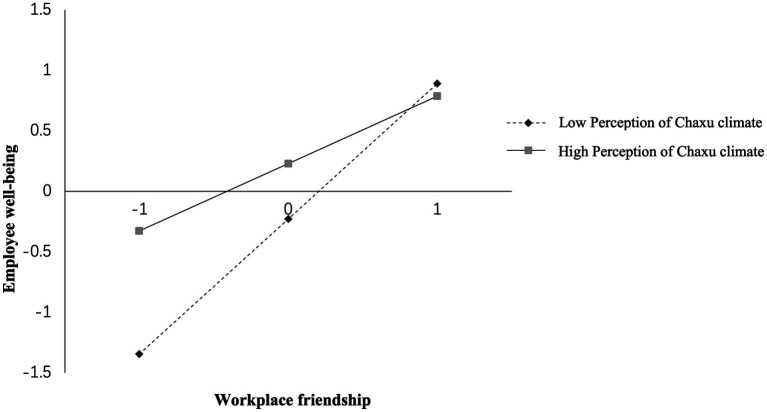
The moderating effect of perception of Chaxu climate on workplace friendship and employee well-being.

Moderating effect test. In order to reduce the influence of multicollinearity on the results, we standardized the variables before calculating the interaction terms. From Table 5, the interaction term between perception of Chaxu climate and workplace friendship negatively affects employee well-being (*β* = −0.192, *p* < 0.001) with a 95% CI of [−0.244, −0.141] and 95% confidence interval excluding 0. This indicates that the stronger the perception of Chaxu climate, the weaker the positive relationship between workplace friendship and employee well-being, which supports H3. And the effect of workplace friendship on employee well-being at different levels of perception of Chaxu climate is shown in Figure 2.

**Table 5:** Interactive effect.

Independent variavles	Dependent variables	Effects	SE	95% CI		*p*
				LL	UL	
WF * CX	EW	−0.192***	0.026	−0.244	−0.141	0.000
	KH	0.103***	0.047	−0.229	−0.283	0.000

Testing for moderated mediating effects. This study followed the method recommended by Edwards and Lambert (2007) to examine the moderated mediating effect played by perception of Chaxu climate. As can be seen in Table 6, the indirect effect of workplace friendship on knowledge hiding reaches the significant level for both high (*β* = 0–0.204, *p* < 0.001) and low (*β* = 0–0.410, *p* < 0.001) perception of Chaxu climate with 95% confidence intervals that do not contain 0. So, H4 is supported by the data.

**Table 6:** The moderated mediating effect.

Level	Paths	Effects	SE	Boot 95%CI		*p*
				LL	UL	
Low CX	Total effect: WF → EW	0.860***	0.059	0.744	0.976	0.000
	Direct effect: WF → HK	−0.274**	0.128	−0.526	−0.022	0.034
	Indirect effect: WF → EW → HK	−0.410**	0.130	−0.616	−0.089	0.000
High CX	Total effect: WF → EW	0.428***	0.035	0.359	0.498	0.000
	Direct effect: WF → HK	−0.041	0.071	−0.181	0.099	0.561
	Indirect effect: WF → EW → HK	−0.204**	0.086	−0.367	−0.031	0.000

## Discussion

### Conclusion

Drawing on social exchange theory (SET), we explore how and when workplace friendship inhibits knowledge hiding from the perspective of employee’s reciprocity. Using data from a two-wave time-lagged survey of 279 employees in China, the results show that workplace friendship positively impacts employee well-being and subsequently, negatively impacts knowledge hiding, i.e., employee well-being plays a mediating role between workplace friendship and employee well-being. Furthermore, employees’ perception of Chaxu climate moderates the direct effect of workplace friendship on employee well-being and the indirect effect of workplace friendship on knowledge hiding *via* employee well-being. As the level of employee’s Perception of Chaxu climate rises, the direct effect of workplace friendship on employee well-being is stronger, so as the indirect effect of workplace friendship on knowledge hiding, and vice versa.

### Theoretical contributions

This study makes several contributions to the existing literatures. First, we contribute to the study on antecedent variables of knowledge hiding by exploring the effect of workplace friendship on employees’ knowledge hiding. Most of the previous articles focus on formal organizational relationships or negative interpersonal factors (Anand et al., 2021; He et al., 2021), while ignoring informal interpersonal relationships, i.e., workplace friendship. For example, Zhao et al. (2016) found that workplace ostracism can increase employees’ tendency to conduct knowledge hiding; and formal leader-member exchange relationships (LMX) in organizations can inhibit knowledge hiding (He et al., 2020). Therefore, our empirical study confirm that the reciprocal psychology induced by workplace friendship through the satisfaction of employees’ job achievements and affective needs motivates them to adopt positive reciprocal behaviors for rewarding the firm/colleagues, which inhibits the occurrence of knowledge hiding.

Second, our study shows that workplace friendship negatively affects employees’ knowledge hiding, which extending the research related to workplace friendship. Previous studies have argued that workplace friendship plays a central role in individual’s work and life. As an informal interpersonal relationship in organizations, workplace friendship provides many advantages for employees. It not only offers them with instrumental and affective support for their development (Zhang et al., 2022), but also benefits teams and organizations by promoting cooperation and unity among colleagues (Xiao et al., 2020). Although workplace friendship has some obvious benefits, increasing research suggests that it may also have complex and negative effects. For example, workplace friendship may decrease individual performance by distracting employees from their focuses and task concentration (Pillemer and Rothbard, 2018). Our findings support that workplace friendship has a positive impact in organizations, i.e., it is positive to employee well-being. This is consistent with the argument that workplace friendship has a positive side and the empirical evidence that friendship is strongly correlated with positive reciprocity (Zhang et al., 2022). On the other hand, although previous studies have examined the effects of workplace friendship on employee positive behavior such as organizational citizenship behavior (Scott et al., 2018), and knowledge sharing (Enwereuzor et al., 2022), the attention has been focused on positive work behaviors. Few have focused on the impact of workplace friendship on the negative interpersonal behavior that is knowledge hiding (Zhuang et al., 2020). By empirically examining the role of workplace friendship in enhancing employee well-being and clarifying the relationship between employee well-being and knowledge hiding, our study advances the previous literature.

Third, our study helps to identify the antecedent of employee well-being and the inhibitory effect of this positive subjective experience on employees’ negative behavior, thus contributing to the literature on employee well-being. For employees, high well-being not only promotes strong positive behavioral tendencies and reduces the negative workplace behaviors, but also improves organizational performance and shapes a win-win situation between employees and the organization through the improvement of their own productivity (Ali et al., 2021). However, its antecedents in the aspects of workplace climate and organizational informal interpersonal are unclear. Although studies have examined the impacts of organizational-level factors on employee well-being (Nielsen et al., 2017), as well as the impacts of high-commitment work systems (Zhang et al., 2022), leadership style (Inceoglu et al., 2018), and supervisor-subordinate relationships (Van Vianen et al., 2022) on employee well-being, there is still a gap in understanding the role of workplace friendship, an informal interpersonal relationship, on the promotion of employee well-being. Furthermore, although the literature has examined the role of employee-related factors in the design and implementation of knowledge management strategies and has emphasized the importance of employee well-being in producing better individual and organizational outcomes, few have investigated Chinese employees’ well-being and their behavioral tendencies to hide knowledge in the context of workplace friendship. Therefore, based on social exchange theory, we explore the mediating role of employee well-being in the path between workplace friendship and knowledge hiding, contributing to the research related to employee well-being.

Fourth, this study explores that perception of Chaxu climate negatively moderates the effect of workplace friendship on knowledge hiding through employee well-being, which contributes to relevant literature of perception of Chaxu climate. On the one hand, previous studies have mainly examined perception of Chaxu climate as an antecedent or outcome factors, few have analyzed the moderating role of it from the perspective of boundary conditions (Huang et al., 2018). The current study explored the moderating role of perception of Chaxu climate to employee well-being and knowledge hiding in a workplace friendship situation, which expands the research of perception of Chaxu climate. On the other hand, as suggested by Peng (2013), it is important to improve the research on the context of knowledge hiding. However, existing research has mainly focused on the organizational level (Men et al., 2020), while less attention has been paid to the boundary conditions of knowledge hiding in terms of individual differences (Zhao and Jiang, 2021). In addition, previous studies of perception of Chaxu climate are usually based on organizational or team, few have explored its impact on employees’ knowledge hiding from individual perceptions (Peng and Zhao, 2011). Therefore, we respond to the call of previous scholars by applying individual perception of Chaxu climate to knowledge hiding and filling the above-mentioned theoretical gap.

### Practical implications

Because flatter organizational structure can facilitate social interactions (Xiao et al., 2020), workplace friendship is more prevalent in current firms. Our study provides several important implications for practice. First, our findings show that workplace friendship has a significant inhibitory effect on employees’ knowledge hiding, indicating that establishing and maintaining friendship is important for the development of employees and organizations. Therefore, we suggest that firms should pay attention to the management of workplace friendship, provide employee with opportunities to establish workplace friendship while providing proper guidance on the direction of workplace friendship and improving the quality of it. Specifically, during the recruitment, managers can conduct some assessment to select employees who can easily get along with others and can further train employees to improve their skills in relation to colleagues through live simulations and role plays. In addition, organizations should improve formal communication channels (such as monthly meetings, seminars, etc.) and informal communication channels (such as e-mail, small gatherings, weekend trips, etc.) among employees to build effective communication mechanisms and platforms for them to form and establish workplace friendships.

Second, our findings demonstrate that employee well-being plays an important mediating role between workplace friendship and knowledge hiding. Therefore, managers should cultivate an environment full of happiness for reducing employees’ psychological stress and interpersonal risks when they provide knowledge. We believe that managers can minimize knowledge hiding by developing an organizational climate of mutual commitment, trust, and positive reciprocity in their organizations. Based on this, managers need to keep an eye on how the organization is managed and try to create a safety environment for employees by providing support in terms of autonomy, etc. These could help to increase the psychological safety of employees when sharing knowledge. In this situation, the satisfaction of employees’ psychological needs can improve the achievement of their self-worth and well-being, which in turn reduces or inhibits the motivation to hinder their colleagues’ access to knowledge (e.g., knowledge hiding). Specifically, organizations can adopt a flexible HR management system that provides employees with quality learning and skill development programs, ensuring that the requirements of the work are compatible with employees’ values and ambitions for meting their psychological needs.

Third, we also found that the effect of workplace friendship on employee well-being was stronger for employees with lower perception of Chaxu climate. Therefore, managers should value this moderating effect for facilitating employee well-being and knowledge sharing by reducing employees’ perception of Chaxu climate. More specifically, managers should allocate organizational resources based on employees’ abilities rather than biased relationships in order to treat each employee as fairly as possible. By creating a fair organizational climate through procedural and information fairness, managers can suppress the expansion of organizational Chaxu climate, enhance employee well-being and motivate employees’ knowledge sharing. In addition, it is worth noting that the “knowledge stocks” of employees varies from person to person. Managers should decrease the perception of Chaxu climate for employees with large “knowledge stocks” through job rotation or multi-team collaboration to reduce knowledge hiding.

### Limitations and future directions

There are still some shortcomings in the followings: First, although this study used a two-wave questionnaire for data collection which improved the repeatability of the findings, there may still exist common method biases since we assess employee well-being and perception of Chaxu climate at the same time. Future research could introduce a longitudinal tracking data collection technique (e.g., empirical sampling method ESM), i.e., a questionnaire survey using “multiple days and multiple points per day” (Lanaj et al., 2014). Furthermore, experimental designs can help to provide more conclusive empirical evidence for causal relationships between variables (He et al., 2020). The scholars could use an experimental design to observe changes in employee well-being and knowledge hiding by manipulating workplace friendship and employee’s perception of Chaxu climate. In addition, in terms of research design, this study only takes gender, age, education level and tenure as the control variables of this research. However, it is worth noting that due to great differences among individuals, workplace friendship may be affected by other factors on employees’ happiness perception and knowledge hiding behavior, such as organizational size and type Employee types (such as knowledge workers and non-knowledge workers) and introversion/extroversion (Xiao et al., 2020). Therefore, more control factors can be considered in future studies to further improve the explanatory power of the model.

Based on social exchange theory, we used employee well-being as a mediating variable to reveal potential mechanism between workplace friendship and knowledge hiding. Future studies can further adopt other mediating variables and theoretical perspectives to deepen the mechanism. For example, according to conservation of resources theory, people have the intention to “retain, protect, and develop resources” (Škerlavaj et al., 2018), and knowledge has long been considered as a vital personal resource, so employees may hide their knowledge for gaining more resource and/or avoiding resource loss. It may be an interesting direction to explain how workplace friendship affect employees’ work resources and their knowledge hiding based on conservation of resources theory. Alternatively, employees’ affections and cognition can be used as a mediating mechanism to explore the effect of workplace friendship on employees’ knowledge hiding from the perspective of affective event theory.

Third, we did not take different dimensions of knowledge hiding (i.e., evasive hiding, playing dumb, and rationalized hiding) into consideration. Among these dimensions, playing dumb and evasive hiding are deceiving, while rationalized hiding is non-deceptive and it is mostly used to protect the benefits of others (Burmeister et al., 2019). For example, Peng (2013) noted that rationalized hiding helps reduce interpersonal risk and stimulates teamwork. Therefore, future research should explore various dimensions of knowledge hiding to find more targeted strategies to address the phenomenon. Furthermore, scholars can advance the research by exploring the unique antecedents of rationalized hiding. Particularly, since employees have motivations to protect information confidentiality or third-party profitability and will be motivated by moral factors when practicing rationalized hiding (Zhao and Xia, 2019), scholars could focus on those factors related to morality (e.g., moral identity, moral disengagement) and individual differences in predicting rationalized hiding among employees who value responsibility and commitment (e.g., organizational commitment).

Fourth, the findings suggest that workplace friendship are correlated to better employee well-being, which confirm the positive effect of workplace friendship. However, although many scholars share the view that workplace friendship leads to desirable organizational outcomes, there may also be a complex and dark side to it due to rapid changes in job responsibilities and social technologies (Pillemer and Rothbard, 2018). For example, workplace friendship may be in trouble if the actions required for employees to achieve instrumental goals conflict with their own social–emotional goals, or if situations such as those excluded from informal groups of colleagues feel marginalized and form their own subgroups, may lead to the emergence of silos and reduced inter-group communication (Carton and Cummings, 2012). So, we suggest that future research could try to explore the impact of workplace friendship on organization and employee by looking at the “dark” side or the “double-edged sword” effect of it.

## Data availability statement

The raw data supporting the conclusions of this article will be made available by the authors, without undue reservation.

## Ethics statement

Ethical review and approval were not required for the study on human participants in accordance with the local legislation and institutional requirements. Written informed consent was provided by participants to participate in this study.

## Author contributions

This study is a joint work of the six authors. PH, JW, and HZ contributed to the ideas of educational research, collection of data, and empirical analysis. CZ, QL, and XX contributed to the data analysis, design of research methods, and tables. PH, JW, HZ, and CZ participated in developing a research design, writing, and interpreting the analysis. All authors contributed to the article and approved the submitted version.

## Funding

Funding was provided by Huaqiao University’s Academic Project Supported by the Fundamental Research Funds for the Central Universities (20SKGC-QT02) and the National Natural Science Foundation of China (72172048).

## Conflict of interest

The authors declare that the research was conducted in the absence of any commercial or financial relationships that could be construed as a potential conflict of interest.

## Publisher’s note

All claims expressed in this article are solely those of the authors and do not necessarily represent those of their affiliated organizations, or those of the publisher, the editors and the reviewers. Any product that may be evaluated in this article, or claim that may be made by its manufacturer, is not guaranteed or endorsed by the publisher.

## Footnotes

^1^ sojump.com
